# A tongue for all seasons: extreme phenotypic flexibility in salamandrid newts

**DOI:** 10.1038/s41598-017-00674-y

**Published:** 2017-04-21

**Authors:** Egon Heiss, Stephan Handschuh, Peter Aerts, Sam Van Wassenbergh

**Affiliations:** 1 Institute of Systematic Zoology and Evolutionary Biology, Friedrich-Schiller-University Jena, Erbertstr. 1, 07743 Jena, Germany; 2 VetCore Facility for Research, Imaging Unit, University for Veterinary Medicine Vienna, Veterinärplatz 1, 1210 Vienna, Austria; 3 Department of Biology, University of Antwerp, Universiteitsplein 1, B-2610 Antwerp, Belgium; 4 Department of Movement and Sports Sciences, Ghent University, Watersportlaan 2, B-9000 Ghent, Belgium; 5 Departement d’Ecologie et de Gestion de la Biodiversité, Muséum National d’ Histoire Naturelle, 57 rue Cuvier, Case postale 55, 75231 Paris Cedex 5, France

## Abstract

Many organisms faced with seasonally fluctuating abiotic and biotic conditions respond by altering their phenotype to account for the demands of environmental changes. Here we discovered that newts, which switch seasonally between an aquatic and terrestrial lifestyle, grow a complex adhesive system on their tongue pad consisting of slender lingual papillae and mucus-producing cells to increase the efficiency of prey capture as they move from water onto land. The adhesive system is reduced again as newts switch back to their aquatic stage, where they use suction to capture prey. As suction performance is also enhanced seasonally by reshaping of the mouth due to the growth of labial lobes, our results show that newts are exceptional in exhibiting phenotypic flexibility in two alternating components (i.e. tongue pad and labial lobes) within a single functional system, and suggest that this form of phenotypic flexibility demands complex genetic regulation.

## Introduction

Phenotypic flexibility describes reversible structural, physiological or behavioral changes within an individual in response to fluctuating environmental conditions^1, 2^ and is hypothesized to be the main factor allowing organisms to adjust to fluctuating environmental conditions through increased performance^3^. Salamandrid newts from the former genus *Triturus* are amongst the most flexible vertebrates by exhibiting a lifestyle in which adults seasonally change between an aquatic and a terrestrial life, exhibiting a distinct morphology in each phase^4–9^. These newts are terrestrial from late summer to early spring, after which they migrate into aquatic habitats for reproduction. After reproduction, the newts leave water and become terrestrial again. This seasonal habitat shift is challenging as functional and physiological demands on the whole organism differ between life in water and on land^10^. One of the main challenges is successful prey capture performance in both environments. Most aquatic vertebrates use suction feeding to capture prey in water^11^. On land, suction feeding is inefficient and the tongue plays a central role in prey capture and intraoral transport^12^. Accordingly, a prey capture system adapted for aquatic strikes is suboptimal for terrestrial ones and vice versa^13^.

Recent studies have shown that newts respond the different functional demands imposed by aquatic and terrestrial feeding with a high degree of behavioral and structural flexibility. Newts use suction feeding in their aquatic stage and tongue prehension in their terrestrial stage. In conjunction with behavioral flexibility, structural flexibility of the prey capture apparatus was also recorded^14–16^. Specifically, labial lobes grow as newts enter their aquatic stage. Labial lobes are skin flaps that grow from the posterior upper jaw to the lower jaw to close the corners of the mouth^16^, forming a tube-like structure^15^, which are later resorbed as the newts leave the aquatic environment. Hydrodynamic simulations recently demonstrated that labial lobes significantly increase suction feeding performance by increasing flow velocities in front of the newt’s mouth by 31%^15^.

This raises the question of whether reversible morphological flexibility of the tongue plays a role in advancing terrestrial feeding. In terrestrial feeding, the tongue accelerates out of the mouth and attaches to prey^7, 17, 18^. The tongue is then retracted and the adhering prey dragged into the mouth. Accordingly, the adhesive capacity of the tongue pad is crucial to terrestrial prey capture and recent studies have shown that amphibians using this mechanism to catch prey have a tongue pad surface studded with surface amplifying slender filiform papillae^19–23^ and abundant mucus producing lingual glands^7, 18^. The interaction of the rough filiform papillae and adhesive lingual mucus is considered important for the adhesive mechanism^21^, improving tongue adhesion. However, an adhesive tongue is useless for suction feeding and specialized suction feeders lack a tongue completely or have a small tongue with a smooth surface and few mucus glands^7, 17, 24–27^.

Analogous to the seasonal appearance and resorption of labial lobes, we hypothesize reversible structural changes in the tongue pad in newts. By using µCT-scanning and graphical 3D reconstructions, quantitative histology and scanning electron microscopy, we test for changes in two functionally and morphologically different systems: dorsal lingual epithelium and lingual sinuses. The dorsal lingual epithelium on the tongue pad is the direct contact zone with the prey in lingual feeding and we hypothesize significant but reversible changes in the tongue pad surface structures (e.g. increase of papillae size and number), increased epithelial thickness and goblet cell density in terrestrial staged newts that advance tongue-to-prey adhesion. Second, we hypothesize that liquid filled sinuses in the anterior tongue might allow volume adjustments of the tongue pad. Lingual sinuses are liquid filled structures in the tongue pad and were first mentioned in newts by Drüner^28^ as “liquid filled lacunae […] that most certainly contribute to lingual movements and play a role in prey grasping” (p. 524; translation by EH). However, the function of the lingual sinuses has never been tested. In analogy to nectar feeding bats that use a hemodynamic mechanism to erect lingual papillae^29^, we hypothesize that the lingual sinuses in multiphasic newts might represent a plastic system that can be engorged and drained by net capillary filtration and by action of contractile elements or valves of the lymphatic system^30, 31^ to increase and decrease tongue pad volume according to functional seasonal demands. Larger and heavier tongues in the newts’ terrestrial stages might be advantageous when tongue is used to catch prey. More specifically, a larger tongue pad may increase the impact of the tongue on the prey and result in a tighter contact between the surfaces of tongue and prey, which in turn might increase adhesive potential of the tongue. A smaller tongue pad, in contrast, allows larger water volumes to be engulfed in aquatic suction feeding strikes, resulting in greater suction feeding performance^12^. In sum, this study will provide new insights into performance increasing phenotypic flexibility in newts that cyclically switch between two very different habitats.

## Results

### Morphology of the tongue pad

**Figure 1:**
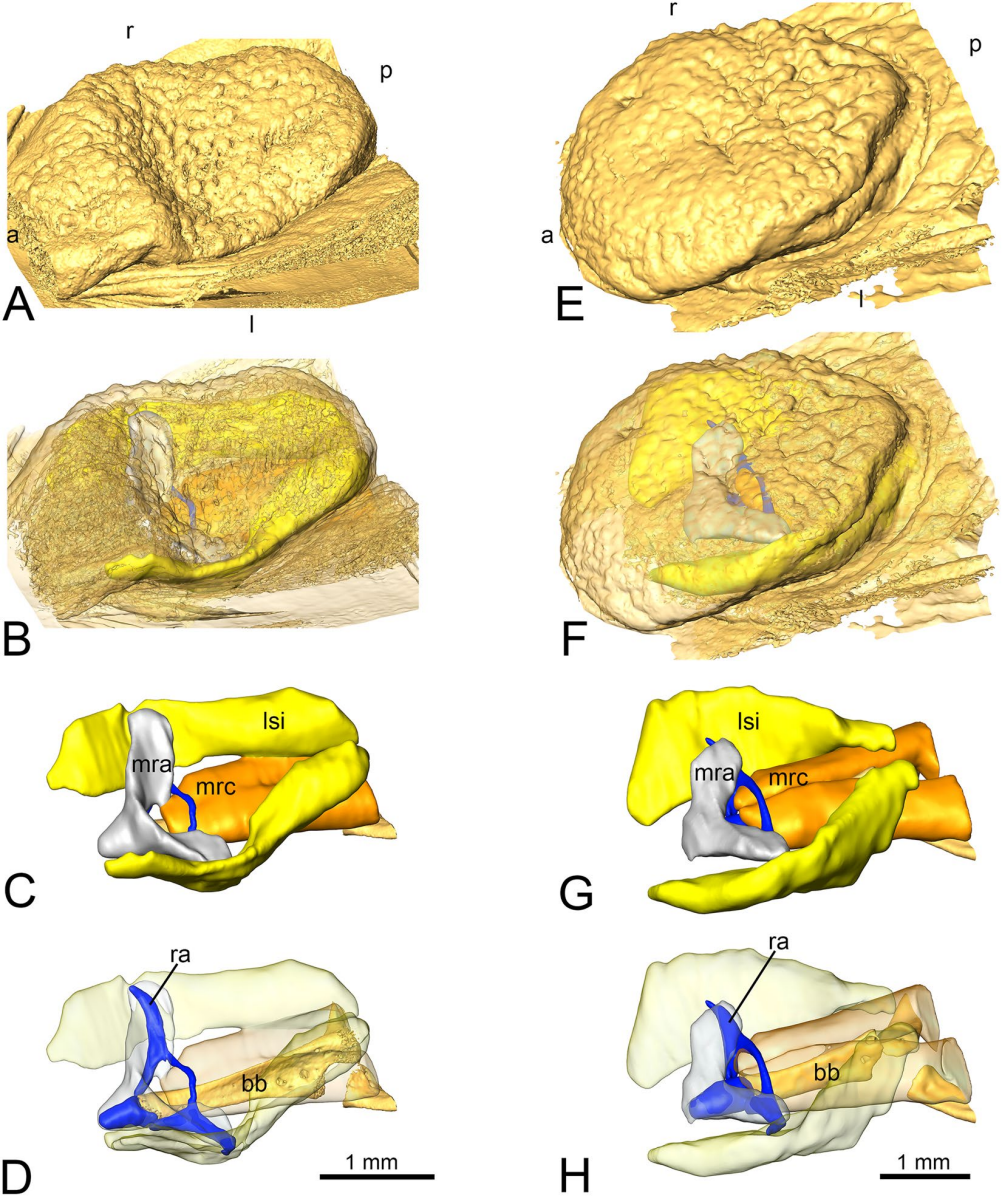
Overview showing the architecture of the tongue pad in the smooth newt *L. vulgaris* ((**A**–**D**), left) and the Alpine newt *I. alpestris* ((**E**–**H**), right) in the aquatic stage. Layers were virtually removed from top to bottom with the top pictures showing the surface of the tongue pad and lower pictures the musculoskeletal and sinuous structures which support the tongue pad system. Abbreviations: (i) Orientation: a, anterior; p, posterior; l, left; r, right. (ii) Anatomical structures: bb, basibranchial; lsi, lingual sinus; mra, radialis muscle; mrc, rectus cervicis muscle; ra, radial cartilage.

The tongue pad in two newt species (*Lissotriton vulgaris*, *Ichthyosaura alpestris*) and both stages (aquatic, terrestrial) appeared as more or less as an oval flap connected anteriorly and ventrally to the floor of mouth (Fig. 1A,E). Beneath the lingual mucosa, two sinuses lay ambilaterally and extended longitudinally from the anterior to the posterior end of the tongue pad (Fig. 1B,C,F,G). Anteriorly, the sinuses were embedded in extensive glandular tissue (tubular lingual glands) restricted to the anterior tongue pad region. More posteriorly, the sinuses were partly enclosed dorsally by the lingual mucosa and ventrally by the rectus cervicis muscle (described below). Acellular condensations were regularly found in the lumen of the sinuses, but no erythrocytes or other cells were evident, pointing to the lymphatic nature of the sinuses. The sinuses were confined by a thin endothelial lining and gave rise to a thin vessel posteriorly. The further course of the vessels that left the sinuses posteriorly could not be traced. Medial to the sinuses, two muscles appeared: anteriorly, the M. radialis and more posteriorly the M. rectus cervicis. The M. radialis ran from the horn-like shaped lingual radii to the anterior tip of the basibranchial (Fig. 1C,G) and the M. rectus cervicis ran from the anterior tip of the basibranchial and the cartilaginous bow connecting left and right radius (Fig. 1C,G) to the first tendinous inscription of the hypaxial musculature (not shown). Next to these two muscles, some fibers of the M. genioglossus fanned out from the anterior dentary into the anterior tongue pad. The medially positioned basibranchial together with the radial system (radii plus bow connecting them), provided skeletal support to the tongue pad (Fig. 1D,H). The radial system was connected through an articulation to the anterior basibranchial.

### Qualitative differences of the dorsal tongue pad surface across stages

**Figure 2:**
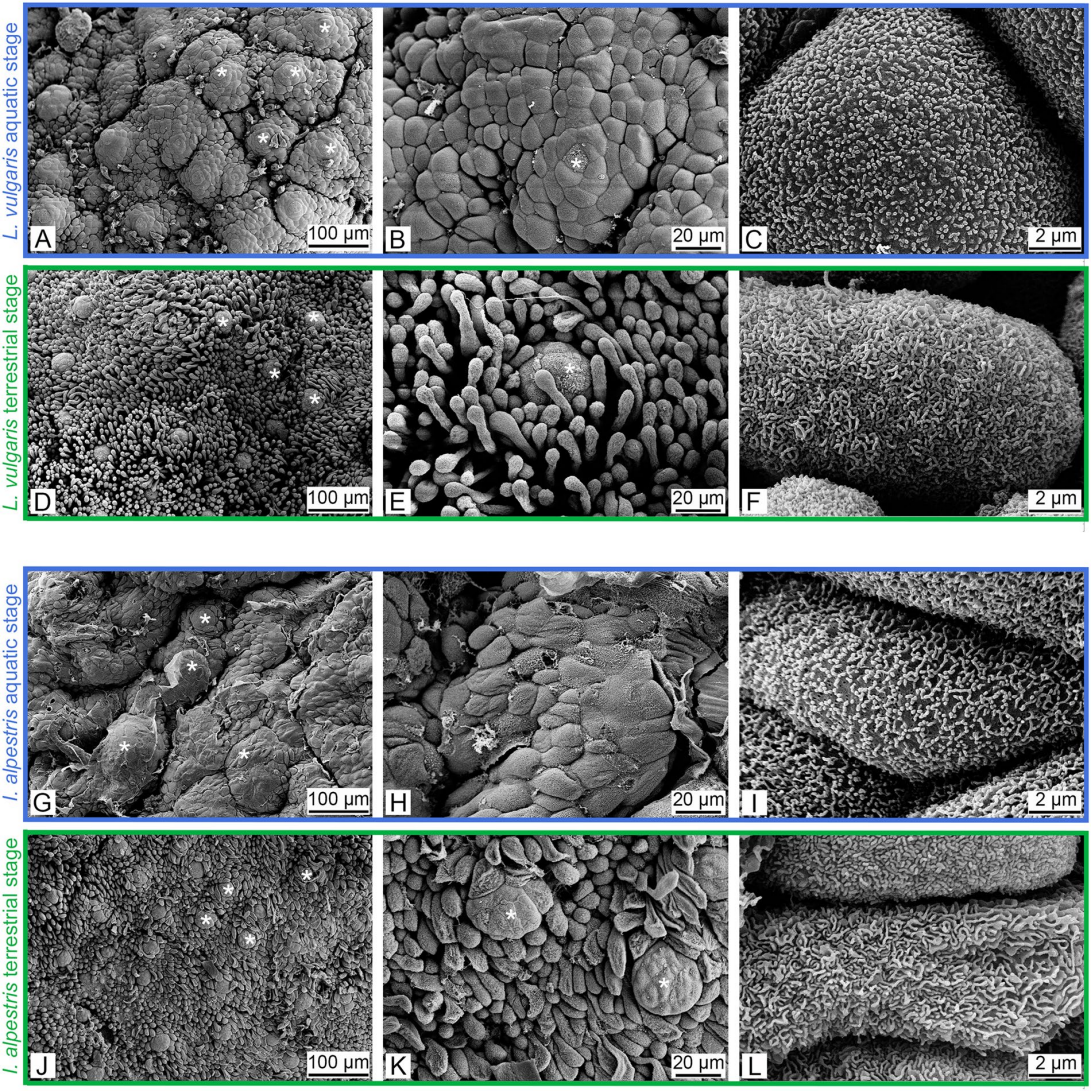
Scanning electron micrographs of the tongue pad surface in *L*. *vulgaris* (**A**–**F**) and *I*. *alpestris* (**G**–**L**) in the aquatic (blue frames) and terrestrial (green frames) stages. Images on the left are overviews and images in the middle and right are higher magnifications. Note the smooth tongue surface in the aquatic stage ((**A**,**B)** *L*. *vulgaris* and (**G**,**H**) *I*. *alpestris*) and the presence of numerous surface-amplifying filiform papillae in the terrestrial stage of both species ((**D**,**E**) *L*. *vulgaris* and (**J**,**K**) *I*. *alpestris*). Taste-bud-bumps (indicated by asterisks) are free-standing in the aquatic stage but surrounded by the filiform papillae in the terrestrial stage. The epithelial cells in the aquatic stage bear microvilli (**C**,**I**), while the surfaces of the slender papillae in the terrestrial stage are covered by rough maze-like microplicae (**F**,**L**).

**Figure 3:**
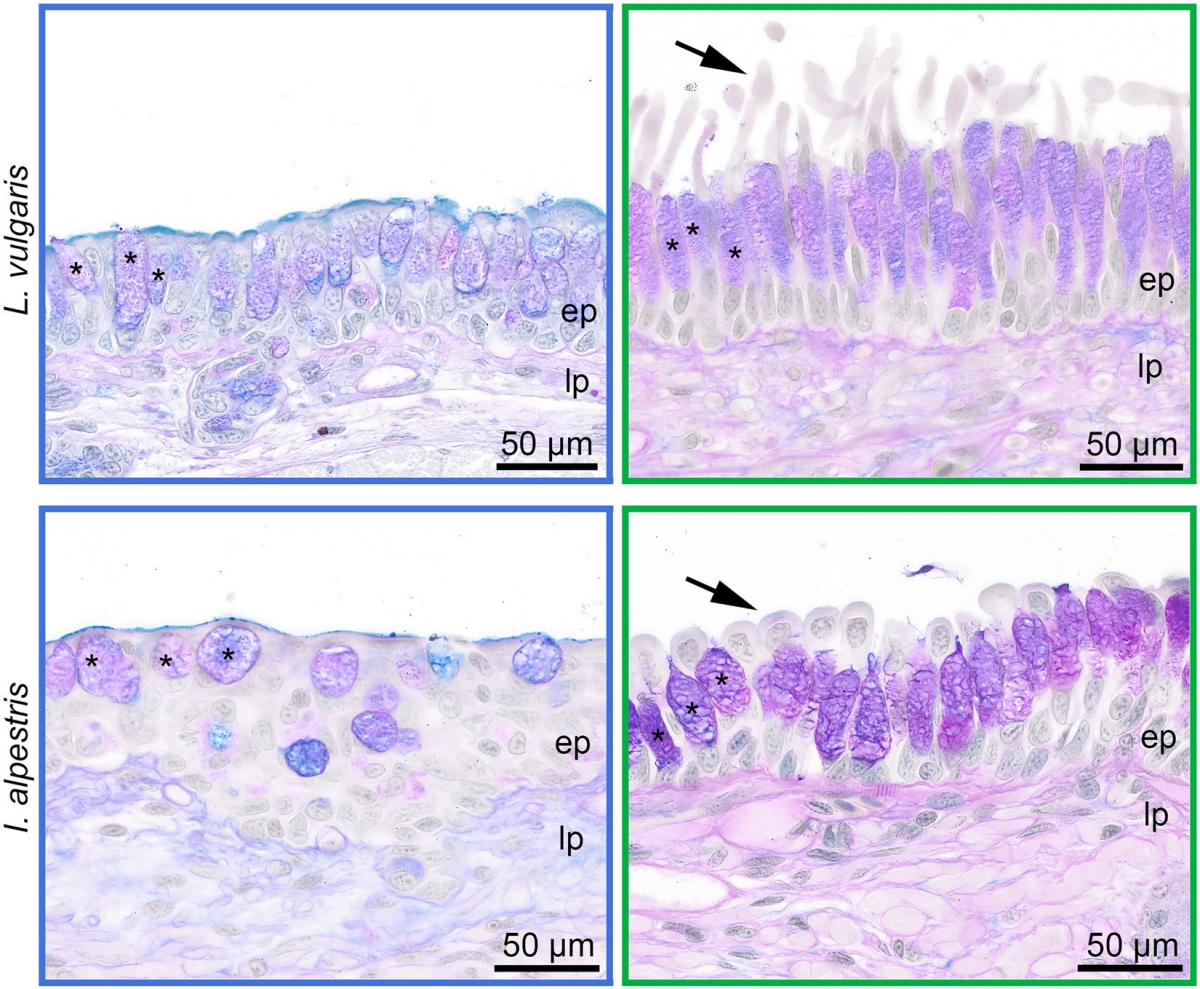
Light micrographs showing the dorsal tongue pad mucosa in the aquatic (blue frames) and terrestrial (green frames) stage in *L*. *vulgaris* (top) and *I*. *alpestris* (bottom). Note the changes in goblet cell number, goblet cell shape (goblet cells indicated by asterisks) as well as presence (terrestrial stage) and absence (aquatic stage) of the slender papillae (indicated by arrows) on the epithelial surface. Abbreviations: ep, epithelium; lp, lamina propria. Alcian blue-PAS staining.

Scanning electron microscopy (SEM) and light microscopy (LM) revealed a relatively smooth surface of the dorsal tongue pad epithelium in the aquatic stage in both newt species (Figs 2A,B,G,H and 3). At low SEM magnification, numerous bumps were visible on the tongue pad, which represented taste buds (Fig. 2A,G). At higher magnification, the cobblestone-like arrangement of the epithelial cells became evident (Fig. 2B,H) and the epithelial cell surface was studded with microvilli (Fig. 2C,I). During the terrestrial stage, the picture changed considerably as filiform papillae covered the epithelial surface and only the tips of the taste-bud bumps were still visible (2D, E, J, K). The filiform papillae were single-celled structures that emerged from the superficial epithelial layer (Fig. 3) and were covered with a complex maze-like arrangement of microplicae, giving the cell surface a rougher appearance compared to the epithelial cells in the aquatic stage (Fig. 2F,L).

### Quantitative differences of the tongue pad across stages

#### Lingual sinus changes across stages

The ANCOVA revealed no significant differences between species (*F*_1,30_ = 0.46; *P* = 0.51), side (*F*_1,30_ = 0.06; *P* = 0.8), or stage (*F*_1,30_ = 0.1; *P* = 0.76) of lingual sinus volume. Similarly, none of the interaction effects showed any significance.

#### Epithelial changes across stages

The Mann-Whitney-U-tests revealed significant differences of epithelial thickness between aquatic and terrestrial stages in *I*. *alpestris*: 70.7 ± 18.4 µm in the aquatic stage vs. 86 ± 17.9 µm in the terrestrial stage (U = 18088; P < 0.001). In contrast, no significant differences of epithelial thickness between aquatic and terrestrial stages were detected in *L*. *vulgaris*: 68.8 ± 14.9 µm in the aquatic stage vs. 67.7 ± 12.4 µm in the terrestrial stage (U = 29267; P = 0.22).

The goblet cell density (number of goblet cells per 100 µm) was significantly higher in the terrestrial stages of both species: 4.15 ± 1.5 (aquatic stage) vs. 6.47 ± 1.31 (terrestrial stage) in *I*. *alpestris* (U = 70; P < 0.001) and 6.21 ± 0.72 (aquatic stage) vs. 8.13 ± 1.17 (terrestrial stage) in *L*. *vulgaris* (U = 60; P < 0.001).

The average shape of goblet cells changed across stages in both species, with goblet cells being high and slender in the terrestrial stage while short and compact in the aquatic stage. The height/width ratio was significantly higher in the terrestrial stage both in *I*. *alpestris* (U = 1525; P < 0.001) (1.66 ± 0.35 in the aquatic stage vs. 2.7 ± 0.67 in the terrestrial stage) and *L*. *vulgaris* (U = 728; P < 0.001) (1.83 ± 0.41 in the aquatic stage vs. 4.27 ± 1.6 in the terrestrial stage).

## Discussion

Our study shows a remarkable phenotypic flexibility in the morphology of the surface of the tongue in newts that seasonally switch between aquatic and terrestrial habitats. In the terrestrial stage, the tongue pad surface becomes studded with numerous slender finger-form papillae that bear an arrangement of rough microplicae (Figs 2 and 3). Furthermore, as the density of goblet cells in the dorsal tongue pad mucosa increases and goblet cells change their shape so that more goblet cells per given surface area can be accommodated, the tongue pad mucosa will be capable of producing more mucus per surface area compared to the aquatic stage. After having transitioned to the aquatic stage, the tongue surface becomes smooth, the epithelium is studded with short microvilli and the mucus producing goblet cells in the lingual epithelium decrease in number (Figs 2 and 3).

These reversible changes to the tongue pad surface when transitioning to the newt’s terrestrial stage improves their performance in capturing prey on land, during which newts predominantly use their tongue: the anterior part of the tongue, with the tongue pad, is accelerated out of the mouth, contacts prey and drags it back into the mouth^14, 17, 32–34^. For an efficient adhesive effect between tongue pad and prey, the tongue pad demands an adhesive system to temporarily “glue” prey^18^. In amphibians, such an adhesion system was recently described in the horned frog *Ceratophrys* sp. and comprises viscous mucus combined with densely packed microscopic slender lingual papillae to function as an adhesive composite^21^. In the terrestrial stage, the tongues of the newts used in this study, with their finger-form filiform papillae bearing a rough surface and increased mucus-producing cells on the tongue pad, fulfill the requirements of such an adhesive composite, similar to the tongue in the horned frog^21, 23^: while the mucus spread in between the filiform papillae serves as the liquid component providing viscous adhesion, the filiform papillae represent the solid component, increasing friction and allowing adaptability of the tongue surface to the surface texture of prey^21, 23^. The rough surface of the filiform papillae both in *L*. *vulgaris* and *I*. *alpestris* might enhance coating of the mucus to the papillae.

In contrast to our initial hypothesis, the large lingual sinuses that run ambilaterally and extend the length of the tongue pad do not change their volume as a consequence of habitat switch. These lymphatic lingual sinuses are thus not engorged in the terrestrial stage or drained in the aquatic stage. But what function can be deduced from these prominent structures? In fact, the lingual sinuses might contribute to lingual prehension by providing a soft and flexible bulge that encircles the free (i.e. lateral and posterior) margins of the tongue pad. During tongue protraction, action of the genioglossus and radialis muscle rotate the tongue pad anteriorly^13, 14, 17, 33^. By rotating the tongue pad anteriorly while protracting the tongue, the medial and posterior parts of the tongue pad are those that hit the prey. The nature of the impact of the tongue on the prey is important for the function of the adhesive system. Kleinteich and Gorb^21, 23^ showed that many frogs have a network of thin, mostly vertically arranged 30 nm thin fibers just beneath the dorsal lingual lining and hypothesized that those fibers, along with filiform papillae, provide a functional cushion that enables the tongue surface to cover uneven surfaces during the impact. In the newts examined here, we suggest that the liquid filled sinuses might exhibit a similar function: providing a soft cushion that enables plastic deformations of the tongue pad during the impact on the target to provide tight contact between tongue and prey. The fact that the sinuses form an elevated bulge that almost completely encircles the tongue pad may suggest a sucker like effect after impact on the target. If true, the tongues of terrestrial newts might function in an analogous way to the adhesive system recently described for the clingfish *Gobiesox maeandricus* ^35^ which use a sucker-disc with edges studded with filiform structures interspersed with mucus. The increase in lingual epithelial thickness in the terrestrial stage in *I*. *alpestris* (not observed in *L*. *vulgaris*) might be related in providing more mechanical robustness of the lingual lining during impact on the target.

The described anatomy of the tongue surface in the aquatic stage, which becomes smoother and poorer in mucus-producing cells (Figs 2 and 3), may explain the unexpected behavior of the newts observed during previous studies. While newts in the terrestrial stage always used lingual prehension to catch prey on land, newts induced to feed on land during the aquatic stage never used a tongue-based prehension mode, and instead used their jaws to grasp prey out of water^32, 33^. A plausible explanation for this stage-dependent terrestrial prehension mode (lingual vs. jaw prehension) might be the lack of adequate adhesion providing structures (i.e. filiform papillae and abundant goblet cells) on the tongue pad in the aquatic stage which makes the tongue as prehensile tool useless.

To our knowledge, the phenotypic flexibility of the prey-capture system in newts is the first example in nature where two separate components of a single functional system, the tongue pad surface and the labial lobes, become alternatingly developed and reduced periodically, correlated by the annual shift of habitat and lifestyle. In the aquatic stage, when newts predominantly use suction feeding, the labial lobes which close the mouth corners, significantly increase suction feeding performance^15^. As newts change to the terrestrial stage, the labial lobes are reduced^16^. This freeing of the corners of the mouth by resorption of the labial lobes is assumed to be beneficial as it will allow the gape to be opened wide enough for the unobstructed tongue projection and subsequent capture and manipulation of large active terrestrial prey with the jaws^15, 36^. The phases of growth and reduction of the labial lobes and the tongue pad’s adhesive structures are thus opposite, and each increases the prey-capture performance in the stage at which they are grown. The most popular examples of phenotypic flexibility in vertebrates in response to environmental fluctuations certainly include seasonal fur and plumage changes in mammals and birds that live in temperate climate zones^37–42^. Less known examples might include songbirds that seasonally grow and shrink song control nuclei in their brains^43–45^, migratory bats and birds that alter organ-, connective tissue- and muscle mass to cope with the energy demands of long-distance migrations^46, 47^, or mammals, sauropsids, amphibians and actinopterygians that undergo structural and physiological changes of the digestive system as response to fluctuating quality and quantity of available food^3, 48–52^. These examples of phenotypic plasticity involve annually only one phase of organ development and one phase of reduction per function, and thus no two, alternating phases of development as for the tongue pad and labial lobes to aid feeding in newts.

Compared to these examples in other vertebrates, the amount of phenotypic flexibility in newts is exceptional. Multiphasic newts go much further and undergo considerable reversible structural, behavioral and physiological changes when cyclically switching habitats. Specifically, when changing from the terrestrial to the aquatic habitat, newts grow tail fins and in the males of some species dorsal crests and foot webs on the hind limbs^4, 6, 53, 54^, the keratinous layers of the skin are reduced^5^, the goblet cells of the nasal mucosa decrease in number and the olfactory cilia become shorter in length^9^, labial lobes grow^16^ while the tongue pad mucosa loses its adhesive potential as the filiform papillae are lost and goblet cell density decreases (this study). In terms of behavior, newts change from quadrupedal locomotion to predominantly undulatory swimming^55^ and from lingual prehension to suction feeding^16, 32, 33, 56^. Physiological changes comprise, amongst others, increase of standard metabolic rates^57^, decrease of osmotic permeability and decrease of active sodium transport across the skin^58, 59^ and reactivation of the lateral line system after being deactivated in the terrestrial stage^60^. All these changes are reversed as newts switch back to terrestrial life. To our best knowledge, newts are the only vertebrates that are capable of such dramatic physiological and morphological reversible cyclic changes as adults.

The final question that arises is how the seasonal phenotypic changes in newts are controlled and regulated. Morphological changes, though season-specific, are not innately timed and phenotypic changes can be triggered by forcing animals into an aquatic or terrestrial lifestyle^9, 54^. This points towards intrinsic control of phenotypic changes as response to changed external environmental circumstances. Seasonal adjustments of so many functional systems might demand complex molecular regulatory mechanisms. Interestingly, salamanders have the largest genome size amongst tetrapods and the average salamander C-value (size of the haplotype) is about ten times larger than that of humans^61, 62^. The question why salamanders have such a large genome compared to other organisms has not been answered adequately yet and this phenomenon was referred to as the “C-value paradox”^63^ or “C-value enigma”^64^. However, recent studies have shown that the large genome size of salamanders is mainly due to exceptionally long introns (non-coding sequences within a gene) in the genome. Historically, these introns were regarded as genetically inert but they might be far more important to the evolution and functional repertoire of complex organisms than has been previously thought^65^. Taft and Mattick^65^ showed that increase of the ratio between non-coding DNA and total DNA content is positively correlated with organismal complexity. Noncoding sequences were suggested to play a major role in the genomic programming, which in turn might result in different levels of biological complexity^65^. More recently, it was suggested that the large introns typical for salamanders may “harbor novel coding- and non-coding sequences that regulate biological processes that are unique to salamanders”^66^. In fact, introns tend to be longer in genes with tissue specific or developmentally relevant functions which may reflect the evolution of complex transcriptional regulatory mechanisms in salamanders^66^. Such regulatory novelties, for example, were related to the extraordinarily developed capability of complex tissue regeneration (e.g. entire limbs after amputation) in salamanders^66, 67^ that demands complex transcriptional regulatory mechanisms. In analogy to tissue regeneration, we hypothesize that similar regulatory mechanisms might be involved in the control of phenotypic flexibility in multiphasic newts. It is therefore not unlikely that the high phenotypic flexibility in seasonal habitat changing newts is regulated by similar complex genetic trajectories that control tissue regeneration, which in turn are related to long introns and large genome sizes.

## Material and Methods

Twenty-four adult Alpine newts (*Ichthyosaura alpestris*) and twenty-four adult smooth newts (*Lissotriton vulgaris*) were collected in their aquatic stage between May 2011 and June 2012 in Lower Austria (Austria) and South Tyrol (Italy) with collection permissions RU5-BE-18/022-2011 (Lower Austria) and 63.01.05/120963 (South Tyrol) granted by the local governments of Lower Austria and South Tyrol, respectively. Twelve individuals for both species were immediately killed and fixed as described below to preserve their aquatic morphotype. The remaining animals were kept in two 150 L tanks with water levels of 15 cm and an easily accessible terrestrial section. Food was offered both in water and on land, and animals were fed twice a week with a variety of red mosquito larvae (chironomids), firebrats (*Thermobia domestica*) and maggots (*Lucilia* sp.). Forty days after each individual newt had left the water and changed to the terrestrial habitat, it was anesthetized and killed in 0.5% aqueous MS222 solution, cut in two pieces approximately 10 mm posterior to the shoulder girdle and immersed into fixation solution as described below. Individual mass was measured before death under anesthesia using a AS60 precision balance (Ohaus, Germany). Animal husbandry and experiments were in strict accordance with national and international laws. All methods were approved by the Ethical Commission for Animal Experiments of the University of Antwerp (code: 2010-36). All procedures were conducted in accordance with their guidelines.

### Histology

For histology, samples of ten adult female *L*. *vulgaris* (five individuals in the aquatic, five in the terrestrial stage) and ten adult female *I*. *alpestris* (five individuals in the aquatic, five in the terrestrial stage) were immersed in Bouin’s decalcifying fixative solution^68^ for two months, changing the solution once a week. After decalcification, the lower jaw including the floor of the mouth and tongue was removed in twelve samples (three individuals per species and stage), dehydrated in a graded ethanol-isopropanol series and embedded in paraffin. In the remaining samples, the entire head (including the lower jaw) was dehydrated as described above and embedded in paraffin. Next, 7 µm semi serial-sections were made on a Reichert-Jung 2030 (Reichert-Jung, Bensheim, Germany) and a MH 360 (Zeiss, Germany) rotatory microtome. The sections were mounted on glass slides and, after removing the paraffin, stained with Azan, periodic acid Schiff (PAS) and a combination of hematoxylin-eosin (HE) according to standard protocols after Böck^68^ and Kiernan^69^. The sections were documented by using a digital camera mounted to an Axiolab microscope (Carl Zeiss Jena, Germany).

### Scanning electron microscopy (SEM)

For SEM, four *L*. *vulgaris* (one female and one male in the aquatic stage, one female and one male in the terrestrial stage) and four *I*. *alpestris* (one female and one male in the aquatic stage, one female and one male in the terrestrial stage) were used. After anesthesia and euthanasia as described above, the floor of the mouth was removed and fixed in modified Karnovsky solution (2.5% glutaraldehyde, 2% formaldehyde in 0.1 M cacodylate buffer with 2% sucrose) for 18 hours^70^. After fixation, samples were rinsed and postfixed in 1% osmium tetroxide for 2 hours, rinsed in distilled water and immersed for 30 minutes in 25% HCl at 40 °C in a Falcon tube, agitating the tube slightly to wash the mucus from the surface. The samples were then rinsed in distilled water and dehydrated in a graded ethanol series, immersed in 100% acetone and critically point dried (Emitech K850 critical point dryer). Next, samples were coated with gold in a sputtercoater (Emitech K500) and analysed in a XL 30 ESEM scanning electron microscope (Philips, Eindhoven, Netherlands).

### Computed Tomography and 3D reconstruction

For µCT scanning, ten male *L*. *vulgaris* and ten male *I*. *alpestris* were fixed in 4% formaldehyde for one month. Then, specimens were dehydrated in a graded series of ethanol. In order to increase x-ray density of soft tissues, specimens were contrasted in a solution of 1% elemental iodine in absolute ethanol for two weeks. After staining, samples were rinsed in absolute ethanol for several hours and mounted in Falcon tubes in absolute ethanol. A scan of the whole head was acquired using a SkyScan 1174 microCT scanner (Bruker microCT, Belgium) with a source voltage of 50 kV and a voxel resolution of 7.39 µm. For a more detailed analysis of the musculoskeletal system of the tongue, a smaller field of view of the region of interest was scanned without previous removal of surrounding tissues. These high-resolution scans of the tongue were made using an XRadia MicroXCT-200 at a source voltage of 40 kV and 2x objective magnification, yielding a voxel resolution of 4.55 µm.

After image acquisition, image stacks were imported into the 3D software package Amira (FEI Visualization Sciences Group, Merignac Cedex, France). Based on tomographic image data, relevant structures were segmented either manually (cartilage, muscles, sinuous volumes) or by threshold segmentation (bones), and visualized via surface renderings.

Recent studies have shown that dehydration using ethanol and iodine staining can result in tissue shrinkage^71^ but given that in the present study all samples were treated the same way, it is highly unlikely that tissue preparation for µCT has influenced inter-individual comparisons of lingual sinus volumes.

### Statistics/measurements

To test for volume differences of lingual sinuses between aquatic and terrestrial stages in the two newt species we measured the volume of left and right lingual sinus in all twenty µCT scanned individuals (five individuals per stage and species). After positively testing for normal distribution of the variables’ residuals (residuals were calculated for each of the independent variables in the ANOVA and the means of each combination of the fixed factors were used to generate the values of the residuals) and homogeneity of the dependent variables, an analysis of covariance (ANCOVA) was performed where sinus volume represented the dependent variable and species (*I*. *alpestris*, *L*. *vulgaris*), side (left, right) and stage (aquatic, terrestrial) were treated as fixed factors and weight as co-factor. By entering the interaction effect of weight and stage into the ANCOVA, different effects (regression coefficient of body mass between stages) of weight and stage were also modeled.

From histological sections, the following parameters were measured from the dorsal tongue pad epithelium: (i) thickness of the epithelium (ii) goblet cell height (iii) goblet cell width (iv) goblet cell density (goblet cell number per 100 µm lingual epithelium). To enable regional comparability, form and position of the hyobranchial musculoskeletal system and overall tongue appearance was used as a reference to ensure that only cross sections from the middle tongue pad region were considered.

Measurements of epithelial thickness (excluding height of filiform papillae) were performed on ten locations on five sections in five individuals of both stages (aquatic, terrestrial) and species (*L*. *vulgaris*, *I*. *alpestris*), resulting in a total of 1000 measurements. The data’s residuals were tested for normal distribution and homogeneity. Normal distribution was not achieved, even after logarithmic transformation of the data. Accordingly, non-parametric Mann-Whitney U tests were performed separately for both species to test for differences between epithelial thickness in aquatic and terrestrial morphs.

To compare shape changes of goblet cells across stages, the ratio of goblet cell height and width was used for further statistical comparisons because height/width ratios provide useful descriptions of the ellipsoid structures of goblet cells. Height and width were measured in ten randomly selected goblet cells on three sections per individual (five), stage (two) and species (two), resulting in a total of 1200 measurements (600 ratios). As data’s residuals didn’t meet the requirements for parametric tests, non-parametric Mann-Whitney U tests were performed separately for both species to test for differences of goblet cell ratios between stages.

Goblet cell density was estimated by counting the total goblet cell number in five sections per individual (five), stage (aquatic, terrestrial) and species (*I*. *alpestris*, *L*. *vulgaris*) and then calculating the average amount of goblet cells per 100 µm epithelium, resulting in a total of 100 measurements. To account for the winding lingual epithelial course, the length of the basal lamina of the dorsal tongue pad epithelium was digitally measured using the measure tool kit of the vector based software InkSkape. As data’s residuals didn’t meet the requirements for parametric tests, non-parametric Mann-Whitney U tests were performed separately for both species to test for differences of goblet cell densities between stages.

## References

[1] Piersma T, Drent J (2003). Phenotypic flexibility and the evolution of organismal design. Trends Ecol Evol.

[2] Piersma T, Lindström Å (1997). Rapid reversible changes in organ size as a component of adaptive behaviour. Trends Ecol Evol.

[3] Naya DE, Farfán G, Sabat P, Méndez MA, Bozinovic F (2005). Digestive morphology and enzyme activity in the Andean toad *Bufo spinulosus*: hard-wired or flexible physiology?. Comp Biochem Phys A.

[4] Dennert W (1924). Über den Bau und die Rückbildung des Flossensaums bei den Urodelen. Anat Embryol.

[5] Warburg M, Rosenberg M (1997). Ultrastructure of ventral epidermis in the terrestrial and aquatic phases of the newt *Triturus vittatus* (Jenyns). Ann Anat.

[6] Brossman K, Carlson B, Swierk L, Langkilde T (2012). Aquatic tail size carries over to the terrestrial phase without impairing locomotion in adult Eastern Red-spotted Newts (*Notophthalmus viridescens viridescens*). Can J Zool.

[7] Fahrenholz, C. In *Handbuch der vergleichenden Anatomie der Wirbeltiere* (eds Bolk, L., Goppert, E., Kallius, E. & Lubosch, W.) 115–210 (Urban und Schwarzenberg, 1937).

[8] Bell G (1977). The life of the smooth newt (*Triturus vulgaris*) after metamorphosis. Ecol Monogr.

[9] Matthes E (1927). Der Einfluss des Mediumwechsels auf das Geruchsvermögen von. Triton. J Comp Physiol.

[10] Denny, M. W. *Air and water: the biology and physics of life’s media* (Princeton University Press, 1993).

[11] Lauder, G. V. In *Functional vertebrate morphology* (eds Hildebrand, M., Bramble, D. M., Liem, K. F. & Wake, D. B.) 210–229 (Harvard University Press, 1985).

[12] Bramble, D. M. & Wake, D. B. In *Functional vertebrate morphology* (eds Hildebrand, M., Bramble, D. M., Liem, K. F. & Wake, D. B.) 230–261 (Harvard University Press, 1985).

[13] Deban, S. In *Vertebrate biomechanics and evolution* (eds Gasc, J. P., Casinos, A. & Bels, V.) 163–180 (BIOS Scientific Publishers, 2003).

[14] Heiss E, Handschuh S, Aerts P, Van Wassenbergh S (2016). Musculoskeletal architecture of the prey capture apparatus in salamandrid newts with multiphasic lifestyle: does anatomy change during the seasonal habitat switches?. J Anat.

[15] Van Wassenbergh S, Heiss E (2016). Phenotypic flexibility of gape anatomy fine-tunes the aquatic prey-capture system of newts. Sci Rep.

[16] Matthes E (1934). Bau und Funktion der Lippensäume wasserlebender Urodelen. Z Morphol Oekol Tiere.

[17] Özeti N, Wake DB (1969). The morphology and evolution of the tongue and associated structures in salamanders and newts (family Salamandridae). Copeia.

[18] Roth G, Wake DB (1985). Trends in the functional morphology and sensorimotor control of feeding behavior in salamanders: an example of the role of internal dynamics in evolution. Acta Biotheor.

[19] Iwasaki SI, Wanichanon C (1993). An ultrastructural study of the dorsal lingual epithelium of the crab‐eating frog, *Rana cancrivora*. J Morphol.

[20] Iwasaki S, Wanichanon C (1991). Fine structure of the dorsal lingual epithelium of the frog, *Rana rugosa*. Tissue Cell.

[21] Kleinteich T, Gorb SN (2015). Frog tongue acts as muscle-powered adhesive tape. R Soc Open Sci.

[22] Opolka A, Wistuba J, Clemen G (2001). The secondary tongue of *Salamandra salamandra*: histochemical and ultrastructural aspects of the developing lingual epithelium. Ann Anat.

[23] Kleinteich T, Gorb SN (2016). Frog tongue surface microstructures: functional and evolutionary patterns. Beilstein J Nanotechnol.

[24] Carreño CA, Nishikawa KC (2010). Aquatic feeding in pipid frogs: the use of suction for prey capture. J Exp Biol.

[25] Beisser CJ, Weisgram J, Splechtna H (1995). Dorsal lingual epithelium of *Platemys pallidipectoris* (Pleurodira, Chelidae). J Morphol.

[26] Lemell P, Beisser CJ, Weisgram J (2000). Morphology and function of the feeding apparatus of *Pelusios castaneus* (Chelonia: Pleurodira). J Morphol.

[27] Deban, S. M. & Wake, D. B. In *Feeding: form*, *function and evolution in tetrapod vertebrates* (ed. Schwenk, K.) 65–94 (Academic, 2000).

[28] Drüner L (1904). Studien zur Anatomie der Zungenbein-, Kiemenbogen- und Kehlkopfmuskeln bei Urodelen. II. Theil. Zool Jahrb Abteil Anat.

[29] Harper CJ, Swartz SM, Brainerd EL (2013). Specialized bat tongue is a hemodynamic nectar mop. P Natl Acad Sci USA.

[30] Francis, E. *The anatomy of the salamander* (Clarendon Press, 1934).

[31] Hedrick MS, Hillman SS, Drewes RC, Withers PC (2013). Lymphatic regulation in nonmammalian vertebrates. J Appl Phys.

[32] Heiss E, Aerts P, Van Wassenbergh S (2013). Masters of change: seasonal plasticity in the prey-capture behavior of the Alpine newt *Ichthyosaura alpestris* (Salamandridae). J Exp Biol.

[33] Heiss E, Aerts P, Van Wassenbergh S (2015). Flexibility is everything: prey capture throughout the seasonal habitat switches in the smooth newt *Lissotriton vulgaris*. Org Divers Evol.

[34] Wake, D. B. & Deban, S. M. In *Feeding: Form*, *Function and Evolution in Tetrapod Vertebrates* (ed. Schwenk, K.) 95–116 (Academic, 2000).

[35] Wainwright DK, Kleinteich T, Kleinteich A, Gorb SN, Summers AP (2013). Stick tight: suction adhesion on irregular surfaces in the northern clingfish. Biol Lett.

[36] Reilly SM, Lauder GV (1990). Metamorphosis of cranial design in tiger salamanders (*Ambystoma tigrinum*): a morphometric analysis of ontogenetic change. J Morphol.

[37] Montgomerie R, Lyon B, Holder K (2001). Dirty ptarmigan: behavioral modification of conspicuous male plumage. Behav Ecol.

[38] Andersson M (1983). On the functions of conspicuous seasonal plumages in birds. Anim Behav.

[39] Hart J (1956). Seasonal changes in insulation of the fur. Can J Zool.

[40] Harris G, Huppi H, Gessaman J (1985). The thermal conductance of winter and summer pelage of *Lepus californicus*. J Therm Biol.

[41] Boyles JG, Bakken GS (2007). Seasonal changes and wind dependence of thermal conductance in dorsal fur from two small mammal species (*Peromyscus leucopus* and *Microtus pennsylvanicus*). J Therm Biol.

[42] Al-Khateeb A, Johnson E (1971). Seasonal changes of pelage in the vole (*Microtus agrestis*): I. Correlation with changes in the endocrine glands. Gen Comp Endocr.

[43] Nottebohm F (1981). A brain for all seasons: cyclical anatomical changes in song control nuclei of the canary brain. Science.

[44] Tramontin AD, Perfito N, Wingfield JC, Brenowitz EA (2001). Seasonal growth of song control nuclei precedes seasonal reproductive development in wild adult song sparrows. Gen Comp Endocr.

[45] Tramontin AD, Brenowitz EA (2000). Seasonal plasticity in the adult brain. Trends Neurosci.

[46] McGuire LP, Fenton MB, Guglielmo CG (2013). Phenotypic flexibility in migrating bats: seasonal variation in body composition, organ sizes and fatty acid profiles. J Exp Biol.

[47] Bauchinger U, Wohlmann A, Biebach H (2005). Flexible remodeling of organ size during spring migration of the garden warbler (*Sylvia borin*). Zoology.

[48] Zaldúa N, Naya DE (2014). Digestive flexibility during fasting in fish: a review. Comp Biochem Phys A.

[49] Starck JM, Rahmaan GHA (2003). Phenotypic flexibility of structure and function of the digestive system of Japanese quail. J Exp Biol.

[50] Starck, J. M. In *Physiological and ecological adaptations to feeding in vertebrates* (eds Starck, J. M. & Wang, T.) 175–200 (Science Publishers, 2005).

[51] Naya DE, Bozinovic F (2004). Digestive phenotypic flexibility in post-metamorphic amphibians: studies on a model organism. Biol Res.

[52] Dekinga A, Dietz MW, Koolhaas A, Piersma T (2001). Time course and reversibility of changes in the gizzards of red knots alternately eating hard and soft food. J Exp Biol.

[53] Nöllert, A. & Nöllert, C. *Die Amphibien Europas: Bestimmung*, *Gefährdung*, *Schutz*. (Franckh-Kosmos, 1992).

[54] Walters, P. J. & Greenwald, L. Physiological adaptations of aquatic newts (*Notophthalmus viridescens*) to a terrestrial environment. *Physiol Zool* 88–98 (1977).

[55] Gvoždík L, Van Damme R (2006). *Triturus* newts defy the running-swimming dilemma. Evolution.

[56] Findeis EK, Bemis WE (1990). Functional morphology of tongue projection in *Taricha torosa* (Urodela: Salamandridae). Zool J Linn Soc.

[57] Kristín P, Gvoždík L (2014). Aquatic‐to‐terrestrial habitat shift reduces energy expenditure in newts. J Exp Zool.

[58] Fenoglio C, de Piceis Polver P (1990). Seasonal variations of K+‐p‐nitrophenyl phosphatase activity in the epidermis of the crested newt: A quantitative and ultrastructural study. Ital J Zool.

[59] Lodi G, Biciotti M, Viotto B (1982). Cutaneous osmoregulation in *Triturus cristatus carnifex* (Laur.) (Urodela). Gen Comp Endocr.

[60] Noble, G. K. *The biology of the Amphibia* (McGraw-Hill, 1931).

[61] Gregory, T. R. *The evolution of the genome* (Academic Press, 2011).

[62] Gregory TR (2002). Genome size and developmental complexity. Genetica.

[63] Thomas CA (1971). The genetic organization of chromosomes. Annu Rev Genet.

[64] Gregory T (2001). Coincidence, coevolution, or causation? DNA content, cellsize, and the C‐value enigma. Biol Rev.

[65] Taft RJ, Mattick JS (2003). Increasing biological complexity is positively correlated with the relative genome-wide expansion of non-protein-coding DNA sequences. Genome Biol.

[66] Smith JJ (2009). Genic regions of a large salamander genome contain long introns and novel genes. BMC genomics.

[67] Kragl M (2009). Cells keep a memory of their tissue origin during axolotl limb regeneration. Nature.

[68] Böck, P. *Romeis Mikroskopische Technik* (Urban and Schwarzenberg, 1989).

[69] Kiernan, J. A. *Histological and Histochemical Methods: Theory and Practice* (Oxford University Press, 2003).

[70] Karnovsky M (1965). A formaldehyde glutaraldehyde fixative of high osmolality for use in electron microscopy. J Cell Biol.

[71] Buytaert J, Goyens J, De Greef D, Aerts P, Dirckx J (2014). Volume shrinkage of bone, brain and muscle tissue in sample preparation for micro-CT and light sheet fluorescence microscopy (LSFM). Microsc Microanal.

